# From (Ontario Ministry of Health and Long-Term Care) policy to implementation: A retrospective look at a community-based patient-centered model of care for chronic pain

**DOI:** 10.1080/24740527.2019.1614880

**Published:** 2019-06-03

**Authors:** Angela Mailis, S. Fatima Lakha

**Affiliations:** a Pain & Wellness Center, Vaughan, Ontario, Canada; b University of Toronto, Ontario, Canada

**Keywords:** chronic pain, community-based, interdisciplinary program, patient-centered

## Abstract

**Background**: Chronic pain is one of the most widely recognized, disabling, and expensive health problems in Canada. Interdisciplinary multimodal pain management is effective in helping chronic pain patients lessen symptoms and reclaim functionality, but most patients lack access to such treatments.

**Aim**: The aim of this study was to describe the development and implementation of a publicly funded and patient-centered model of care in the community.

**Methods**: The study was set in the Pain & Wellness Centre (PWC) in Vaughan, the only community-based chronic pain clinic in Ontario funded by the Ontario Ministry of Health and Long-Term Care (MOHLTC) as a demonstration project of a template for similar future community clinics. The study is descriptive, including a brief review of the Ontario comprehensive pain strategy framework and an overview of the PWC and the process involved in the development of an interdisciplinary pain program (IDP), based on the biopsychosocial model of chronic pain management.

**Results**: During a 2.5-year period, the PWC has offered 1055 new patient medical consultations and 1921 follow-up visits and admitted 242 patients in the IDP program (demonstrating significant success in patient outcomes at the 3-month exit from the program). It established robust outcomes research, organized educational programs for pain trainees, and cultivated a collaborative relationship with the Toronto Academic Pain Medicine (TAPMI) network and the community at large.

**Conclusions**: This demonstration program has shown the feasibility and applicability of the principles of the MOHLTC comprehensive pain strategy, providing an effective, evidence-based, and accountable approach to chronic pain diagnosis and management in the community.

## RÉSUMÉ

**Contexte**: La douleur chronique est l’un des problèmes de santé les plus reconnus, invalidants et coûteux au Canada. La prise en charge multimodale interdisciplinaire est efficace pour aider les patients souffrant de douleur chronique à diminuer leurs symptômes et à recouvrer leur fonctionnalité, mais la plupart des patients n’ont pas accès à de tels traitements.

**But**: Le but de cette étude était de décrire l’élaboration et la mise oeuvre d’un modèle communautaire de soins axé sur le patient, financé par des fonds publics.

**Méthodes**: L’étude s’est déroulée au Pain and Wellness Centre à Vaughan, la seule clinique de la douleur chronique communautaire en Ontario. Elle était financée par le MSSLD en tant que projet de démonstration pouvant servir de modèle pour des cliniques communautaires semblables dans l’avenir. L’étude est descriptive et comprend notamment un survol du cadre stratégique global pour la douleur de l’Ontario et un aperçu du Pain & Wellness Centre, de même que du processus menant à l’élaboration d’un programme interdisciplinaire de la douleur fondé sur le modèle bio-psycho-social de la prise en charge de la douleur chronique.

**Résultats**: Au cours d’une période de 2,5 ans, le PWC a offert des consultations médicales à 1055 nouveaux patients et 1921 visites de suivi, en plus d’accueillir 242 patients dans le programme interdisciplinaire de la douleur (tout en démontrant un important succès dans les résultats des patients trois mois après leur sortie du programme). Il a obtenu des résultats de recherche robustes, organisé des programmes de formation pour des stagiaires et cultivé une relation de collaboration avec le réseau du Toronto Academic Pain Medicine, ainsi qu’avec la collectivité dans son ensemble.

**Conclusions**: Ce programme a démontré la faisabilité et l’applicabilité des principes de la stratégie globale du ministère de la Santé et ds Soins de longue durée de l’Ontario en matière de douleur, offrant ainsi une approche communautaire efficace, fondée sur des données probantes, ainsi que sur une approche responsable du diagnostic et de la prise en charge de la douleur chronique.

## Background

### The burden of chronic pain in Canada

Chronic pain is defined as (1) pain lasting >3 to 6 months or (2) beyond the expected healing time typical of a disease or injury. Chronic pain can arise from conditions of muscles, bones, or ligaments (such as pain from arthritis, low back pain, headaches, etc.), damage of the nervous system (from diabetes, shingles, spinal cord injury, nerve injury after surgery or trauma, etc.), disease or damage of viscera (kidney, heart, gut, etc.), or a combination. However, there may also be no clear cause. Generally, chronic pain (most often identified as chronic noncancer pain or CNCP) arises from a multiplicity of heterogeneous and overlapping pain conditions together with influences from psychosocial and environmental factors. Systemic and constitutional problems (such as fatigue, sleep disturbance, decreased appetite, depression and anxiety), often accompany chronic pain and add to disability and impaired quality of life.^1^

Studies of chronic pain in Canada have found that it is a commonly reported condition.^2^ Among people living in private households, 16% of those aged 18 to 64 and 27% of seniors reported chronic pain. The prevalence is greater (up to 38%) for seniors living in long-term health care institutions.^3^ Women, older individuals, and those with low educational attainment are more likely to report chronic pain.^4–6^ Chronic pain is often associated with dependency in activities of daily living (for example, personal care, moving around the home) and instrumental activities of daily living (for example, errands, housework).^7^ People reporting chronic pain are more likely to use medications and be multiple medication users.^3^ The burden of chronic pain is expected to increase as Canada’s population is aging. Statistics Canada^8^ reported that the proportion of the senior population (aged 65 and older) grew from 8% to 14% between 1971 and 2010, and the proportion of seniors is expected to represent between 23% and 25% of the total population in 2036.

Another major issue in Canada relating to chronic pain is the opioid epidemic. Opioid-related deaths in Ontario increased by 285% between 1995 and 2015, from 14 opioid-related deaths per million population in 1991 to 53 per million in 2015.^9^

### Challenges and costs of chronic pain services in Canada

The current system for chronic pain care in Canada leaves numerous patients misdiagnosed, improperly treated or undertreated, and living with poor quality of life.^10^ Chronic pain is often associated with other diseases and is therefore inadequately recorded, both in clinical records and in the administrative coding that is used as a source for epidemiological studies. Undertreated or inappropriately treated chronic pain results in financial burden borne by both the patients and their families and the public health care system. Chronic pain includes various groups of diagnoses and syndromes; therefore, treatment options vary. Treatment of chronic pain needs highly specialized and variable approaches (such as medications, psychological therapies, exercise therapy, injections, spinal cord stimulators, implantable pumps, etc.).^11^ In Ontario (and most or all of the other Canadian provinces), many of these treatment options are paid by third-party payers, extended health benefits, or, for those patients who have none of these options, out of pocket. The publicly funded health care system in Ontario (Ontario Health Insurance Plan [OHIP]) covers medical consultations, laboratory tests, and imaging in general, though injections are the only diagnostic/treatment modality paid by OHIP, resulting in substantial costs to the provincial health care budget. In particular, in Ontario, medical clinics in the community that only provide interventions (injections) are estimated to cost the system approximately $80M yearly, with 6% of patients seen repeatedly over a period of years accounting for 41% of the injection expenditure, totaling $215M for the period 2005–2012, based on OHIP data.^12^ Overall, the costs of chronic pain to Ontario (including Toronto) have been estimated to be more than 2.1 billion a year in direct health care costs and $13 billion per year in productivity costs related to job losses and sick days.^13^

### Ontario comprehensive chronic pain strategy framework

Given the increasing burden of chronic pain coupled with the opioid epidemic, in January 2011 the Ontario Ministry of Health and Long-Term Care (MOHLTC) created a Chronic Pain Working Group, which included members from MOHLTC, the Ontario Medical Association, and the College of Physicians and Surgeons of Ontario. In November 2011, this group produced a white paper reporting on the current state of services available and provided recommendations for future action^24^. The working group pointed to a large number of problems in the province, including lack of: recognition of the magnitude of chronic pain; availability or access to treatment modalities and services; oversight, standardization, and unified policies; inadequate education for both health care providers and patients; strategies for prevention of chronicity; accountability and outcome measures; supports for primary care; and sustainable financing for providers and services. Details of the findings of the working group are outlined in Table 1.

**Table 1.** Findings of the working group 2011 white paper.^24^

Problems with diagnosis and management of chronic pain in Ontario
Lack of recognition and awareness of the magnitude of chronic pain problem; cost (human and monetary); and knowledge of how to diagnose and manage CNCP (by all providers). Lack of treatment modalities and services because effective CNCP programs/services are not readily available or accessible; effective drugs or nondrug modalities also are not available or accessible; lack of directory of services and programs that do exist. Lack of oversight, standardization, and education, namely, lack of unified policy for CNCP; lack of standards for pain programs/clinics; inadequate education and training in CNCP within the undergraduate curricula, postgraduate programs, and continuing health education for practicing professionals; and lack of accreditation for health care providers to deliver CNCP care. Lack of systematic treatment for populations, including all Ontarians, as well as most vulnerable people such as aboriginals, immigrants, elderly in long-term care, addicts with chronic pain, and the military. Lack of prevention services, specifically, lack of strategies to minimize transition from acute to chronic pain management; lack of self-management programs; and no funding of effective vaccine shown to prevent/reduce incidence of shingles/postherpetic neuralgia. Lack of accountability with no organized system able to measure outcomes or conduct research. Scarcity of chronic pain care delivery at the level of primary care resulting from a lack of supportive services for primary health care providers in managing chronic pain; guidelines/care pathways for chronic pain; availability of stepped-up comprehensive continuum of care for patients with chronic pain from primary care up to the tertiary care level; ongoing mentoring and continuing education to primary health care providers; and chronic pain management within integrated models of care at the primary health care provider level. Financial considerations, such as absence of remuneration specifically for managing patients with chronic pain at both a primary and specialty care level (i.e., no fee code for chronic pain care); lack of remuneration for team-based care involving allied health professionals, who are widely used by the public and important for multidisciplinary management; providing funds for treatment modalities that are shown to be ineffective while there is no funding for treatments that have been shown to work.

CNCP = chronic noncancer pain.

The working group subsequently determined the essential elements that would define and support a comprehensive pain system in the province, summarized in 15 vital principles (pillars). The pillars stressed the need for a governance or oversight body for pain policies and services; a patient-centered and primary care–focused approach; interdisciplinary care in the context of a chronic disease management framework; timely and stepped-up care in the care continuum; consistent, accountable, and measurable care with continuous quality improvement; application of evidence-based treatment approaches and measures for prevention of pain and pain chronicity; education for providers and patients; provisions for data information; and provisions for appropriate and sustainable funding. The 15 pillars of the Ontario comprehensive pain strategy are reported in Table 2.

**Table 2.** Pillars of the Ontario comprehensive pain strategy (working group 2011 white paper).^24^

Pillar	Definition
1. Oversight body	Body responsible for providing supervision, policy development, governance structure, monitoring, and reporting
2. Patient focused	Ensuring timely comprehensive assessment and management of the whole patient Ensuring that the patient’s needs and best interests are considered in each step of the care continuum
3. Primary care focused	Ensuring that the cornerstone of chronic pain prevention and management, the primary care physician, is supported and that his or her involvement is reflected in each step of the care continuum
4. Interdisciplinary care	Team-based care The patient is seen by the right practitioner at the right time in the right place
5. Chronic disease management framework	Care for CNCP should be managed within the framework developed to guide the effective prevention and management of chronic diseases
6. Reasonable access to the care continuum	Patients and physicians should be able to receive the care and assistance required in a timely manner
7. Stepped-up care in a care continuum	Self-help Seamless and timely transition from primary care to secondary and tertiary care as needed Develop and update care pathways Interdisciplinary in all stages
8. Continuous quality improvement	Define quality indicators, set system-wide improvement goals, and evaluate progress toward these goals
9. Evidence based	Synthesize best available evidence Set research agenda Create system-wide capabilities for relevant data collection Develop and translate guidelines into useful point of care tools
10. Accountability	Common framework for community and hospital clinics Outcome measures Evidence-based best practices
11. Consistency	Must meet established criteria (providers and centers) Develop unified policies and standards of care Develop standardized education and accreditation for programs, clinics, and providers
12. Prevention and early intervention	Provide prevention and early intervention (by implementing strategies to lessen transition from acute to chronic pain)
13. Education for patients and providers	Provide education for patients and providers (by introducing standardized pain curriculum in undergraduate and graduate training of health providers [medical and nonmedical] and ongoing education for practicing providers on best practices)
14. Data information (registry, research, supports)	Make provisions for data information (registry, research, supports) by underpinning future improvements, development of standards, prevention strategies, etc.; with system organization that provides a knowledge base for research; utilizing technology (telemedicine) to reach underserviced areas; providing warm and hot (telephone) consultation between primary care and pain specialists; and creating a single referral portal
15. Appropriate and sustainably resourced	Appropriately and sustainably resourced (so that providers and clinics have the right funding and resources required)

CNCP = chronic noncancer pain.

### MOHLTC acting on the Ontario comprehensive chronic pain strategy framework

Starting in 2014, MOHLTC provided $18M in base funding to support interdisciplinary chronic pain management across five pediatric hospitals, 13 adult hospitals, and one community chronic pain clinic in the province of Ontario. The funding exclusively supported teams of allied health professionals and administrative support staff to allow more patients to access the biopsychosocial model of care.

This funding is coupled with other key initiatives supporting chronic pain care across the lines of the Ontario comprehensive chronic pain strategy framework, such as the creation of pediatric and adult advisory boards to develop a networked system with a common information registry and standard models of care; Project ECHO (Extension for Community Healthcare Outcomes, which started in 2014), which connects primary care providers from across Ontario with each other and with interdisciplinary pain specialist teams via weekly videoconferencing sessions; integrating patients from the provincial committee level to each individual chronic pain clinic; creating new quality standards by Health Quality Ontario; developing Inter-professional Spine Assessment and Education Clinics (low back pain centers), which started in 2013 and expanded by 2017; continuing with a Narcotics Monitoring System; publicly funding shingles vaccines for people between 65 and 70 years of age (announced in 2016); and promoting an opioid strategy (announced in 2016) that includes chronic pain management as a key component of the strategy (MOHLTC Ontario Chronic Pain Advisory Network Briefing Document [Pediatric and Adult], excerpts from email, February 1, 2018).

The current article reports on the Pain & Wellness Centre (PWC) in Vaughan, the only community-based chronic pain clinic funded by the MOHLTC funding envelope.

## Overview of the Pain and Wellness Centre

The PWC was founded privately by the primary author, a specialist in physical medicine and rehabilitation and professor at the University of Toronto, Faculty of Medicine, with 37 years of experience currently in pain medicine. The PWC started delivering services in the fall of 2014, including medical consultations (on OHIP fee-for-service, FFS) and rehabilitative multimodal interventions (on extended health benefits, third-party payers, and FFS). The PWC mission from its inception was to provide chronic pain consultations and interdisciplinary chronic pain management in the regions of York, Durham, Peel, Dufferin, and Simcoe (an area of 12 000 km^2^ and 3.7 million patients, north of Highway 401). The PWC was funded in 2016 by MOHLTC under the premises that it was a demonstration project and, if successful, could serve as a template for other publicly funded community-based clinics. Once MOHLTC direct funding became available to this clinic, the mission expanded beyond medical management and taxpayer-funded interdisciplinary pain management to include education and research, based on the pillars of the 2011 framework of the Ontario comprehensive pain strategy.

The primary goals of the PWC are to

- provide timely access to pain consultations and evidence-based pain care in the community, resulting in early treatment.
- offer interdisciplinary treatment to eligible patients with chronic pain in the community.
- facilitate stepped-up care (depending on severity and complexity of the problem) to and from the specialized services of the Toronto hospital-based academic pain clinics and other community-based specialized clinics.
- provide community-based chronic pain education to medical and other health science trainees (academic learners), practicing physicians (lifelong learners). and the local public (patients, families. etc.).
- research chronic pain in a community setting with a focus on interdisciplinary pain treatment outcomes and pragmatic studies.

### Clinical setting

The PWC is a free-standing two-story, three-level facility in the city of Vaughan, 45 km north of Toronto central, wheelchair accessible, and occupying 6400 ft^2^ of space. The facility includes reception areas, medical and administrative offices, treatment rooms, a meditation loft, a board room, staff cafeteria and kitchen facilities, a 1200 ft^2^ fully equipped gym, and its own parking lot. It operates as a paperless facility with an electronic medical records system safeguarded by a cloud-based company. The PWC offers medical consultations and management (funded by OHIP), is accessible to community referrals for treatment of patients funded by third parties (such as auto insurance, etc.), and reports directly to MOHLTC, which funds interdisciplinary pain services and support staff.

### Staff

Currently the PWC staff consists of two physicians (both specialists in physical medicine and rehabilitation), one psychologist and one psychotherapist, a mindfulness meditation facilitator, two naturopathic doctors (NDs), a holistic dietician, two massage therapists, four chiropractors, a community resource facilitator (community navigator), two information technology/management staff, four reception staff, one or two research staff, and a physician assistant. Several rotating trainees such as University of Toronto pain fellows, Royal College pain medicine subspecialty residents, as well as physical medicine and family medicine residents, and chiropractic interns obtain education at the PWC. All of our chiropractors are strength trainers and have additional training and expertise in advanced soft tissue therapy techniques, medical acupuncture, concussion management, and athletic sports injuries. Additionally, all are trained as physician assistants for chronic pain consultations. The massage staff has additional training in lymphatic drainage, and two of the staff are certified life coaches. The NDs and holistic dietitian place emphasis on nutrition, weight management, and life choices. The mindfulness meditation program is offered in a small-group format over an 11-week period, 2 h per week. For patients unable to attend these classes, customized programs are offered with one-to-one mindfulness sessions.

The PWC staff is offered access to PWC-funded continuous health education, including presentations and attendance at our annual Canadian Pain Conference meetings.

### Patient flow

Medical consultation requests for assessment and treatment are faxed to the PWC and reviewed by medical and administrative staff within 48 h of receipt to ascertain basic information (in regard to summarized medical history and relevant investigations/consultations). Referrals that lack information are faxed back to referring physicians requesting missing information. Rejected referrals comprise 20% to 40% of referrals, based on internal statistics, with 60% or more returning with additional information.^14^ Once the referral is deemed adequate, it immediately receives a classification based on distance from the PWC (more or less than 50 km from the facility) and complexity (based on forwarded medical information and classified as simple, medium complexity, and very complex). Given our short wait list of 1 to 4 weeks, patients are called shortly after an adequate referral is received and an appointment is given. Automated voice messages are left at the patient’s phone as a reminder 48 h prior to the appointment.

### Consultation service

All patients (whose referrals are considered adequate) receive a 2-h consultation with a physician or a physician in conjunction with a physician assistant (trained chiropractor or other manual therapist), pain fellow, or resident. In addition to history and thorough neuromusculoskeletal evaluation, patients complete validated batteries such as the Brief Pain Inventory (BPI), the Patient Health Questionnaire, the General Anxiety Disorder Questionnaire (GAD-7), and the Rivermead Concussion Scale as proxy measurements for pain interference, depression, anxiety, and multiple systemic and constitutional problems, respectively. In general, about 25% to 28% of referred patients are deemed very complex (with multiple medical and/or psychiatric comorbidities), 50% are of medium complexity, and 22% to 25% are deemed fairly simple (usually musculoskeletal and/or soft tissue problems).

**Figure 1.**
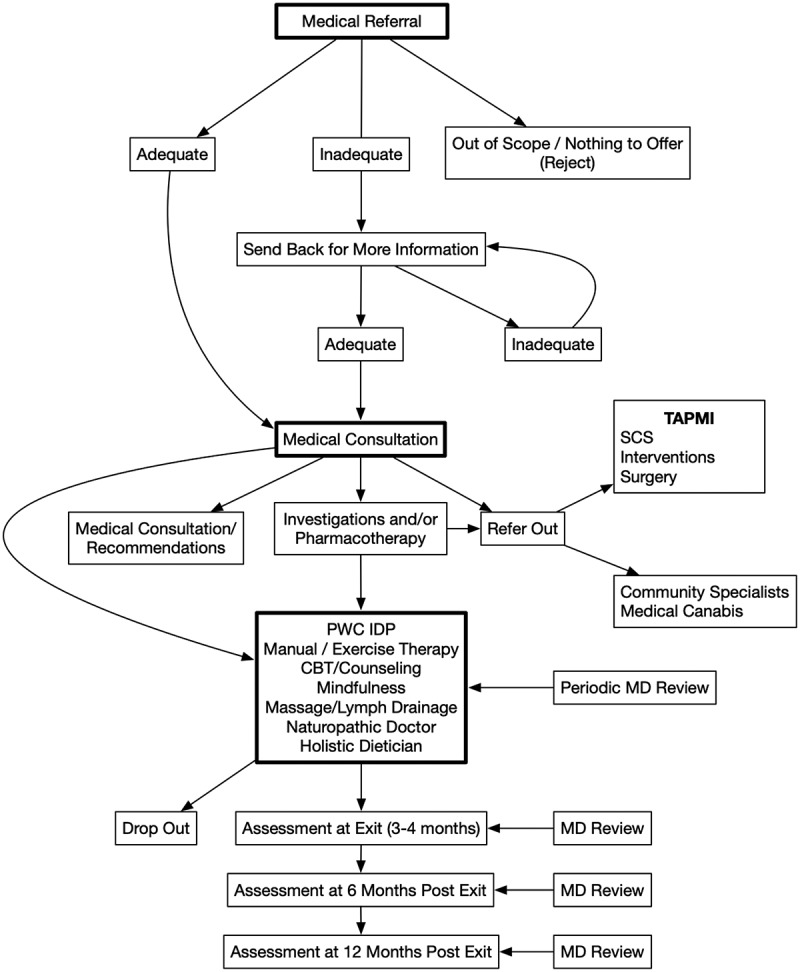
PWC patient flowchart.

Once the patient is seen at the PWC, the physicians may (1) provide single consultation with recommendations for investigations and treatment to a referring physician; (2) order additional investigations and/or initiate/alter pharmacotherapy and arrange for follow-up at the PWC; (3) deem the patient a candidate for the PWC interdisciplinary pain program (IDP) funded by MOHLTC; or (4) refer to external sources/services such as interventions (nerve blocks and infusions), spinal stimulators, surgery (e.g., joint replacement, neurosurgical procedures), medical cannabis, etc. The IDP path can also be accessed once requested investigations are completed or pharmacotherapy (for example, for depression or anxiety, neuropathic pain, etc.) is deemed to be at least partially effective. The patient flow is shown in Figure 1.

Despite our original goal to serve five local regions, referrals with request for consultation are now received from across the entire province of Ontario (and occasionally from other provinces) as far as Sudbury, North Bay, Hamilton, London, Ottawa, Niagara, St. Catharines, etc. Given the very long waiting lists of the downtown academic hospitals, as well as the expertise of our team, we felt compelled to at least offer consulting services to those remote area referrals and advice to referring physicians.

Since MOHLTC funding began as of April 1, 2016, the clinic has medically assessed 1055 new patients and provided 1921 medical follow-up visits in 30 months.

### Interdisciplinary Pain Program

Patients are deemed candidates for IDP if they fulfill the following criteria:

1. Distance from the PWC no more than 50 km to allow for frequent attendance (though exceptions are granted on an individual basis).
2. Inability to afford treatment.
3. Good working knowledge of English because poor English precludes participation in cognitive behavioral therapy (CBT), mindfulness, and nutritional counseling (though occasionally exemptions are made for older patients who are in need of manual/exercise therapy, massage, and nutrition and are capable of bringing a family member to assist with the language).
4. Not experiencing major untreated psychopathology or life crisis.
5. High levels of motivation and commitment.
6. Condition that we feel can be treated in our center.
7. Availability to attend the PWC two to three times a week for a minimum of 2 h at a time for a period of 3 to 4 months.

These criteria were a lot more lenient at the beginning of our program. However, we experienced very high drop-out rates from patients who drove long distances (particularly those who lived in snowbelts north of the Greater Toronto Area), patients with very low functionality and established widespread pain, and patients in major life crises such as being in the midst of an acrimonious divorce, custodial battle, etc., obliging us to narrow our admission criteria.

Approximately 75% to 80% of all patients referred for consultation live within a 50-km radius of the PWC. Of those, 20% to 25% fulfill our eligibility criteria and are offered admission to the IDP.

Once the patient is determined to be a candidate for IDP, he or she participates in a 2-h intake interview with administrative staff, which includes the completion of an explicit consent form (outlining the types of treatments he or she will receive, his or her obligations and commitment to the program, and acceptance to use his or her data anonymously in aggregate format for reporting to the MOHLTC and for research purposes), a tour of the facility, explanations about the services that he or she will receive and take-home explanatory pamphlets. Patients are informed repeatedly by the treating physicians and during consent completion that two or three unjustified no-shows or cancellations may result in program termination. Additionally, at the same intake interview, patients see a research staff to complete a detailed demographic questionnaire and a battery of validated questionnaires, namely, the Pain Catastrophizing Scale; Pain Self-Efficacy Scale; Chronic Pain Acceptance Questionnaire; Tampa Scale for Kinesiophobia; GAD; Centre for Epidemiological Studies–Depression Scale; and BPI. All data are collected electronically and overseen by our staff at the time of the intake interview (our previous method of providing the questionnaires in paper format resulted in faulty and missing data). We also have a significant population of patients who are not sufficiently computer literate to do it on their own on tablets. Nevertheless, we hope to be able to provide direct tablet access to those who are computer literate in the future.

IDP patients are then provided a detailed schedule agreed upon by themselves and an administrative staff for a 3-month period. Each patient receives a minimum of three and up to six services, attending twice a week for a minimum of 2 h. Most patients also attend an 11-week mindfulness group course that includes no more than eight participants. The first appointment with each service provider is devoted to education, outlining specific goals and objectives agreed upon between the provider and the patient and initiating therapeutic intervention. Manual and exercise rehabilitation is the cornerstone of our IDP, passive therapies are used to facilitate introduction to exercise in our gym, and patients are provided with home exercise programs that they review with their chiropractors. Types and frequency of services as well as a typical full-service IDP schedule are shown in Table 3.

**Table 3.** Service provisions at the PWC.^a^

Service distribution	Manual therapy 97% CBT, psychological counseling 69% Mindfulness 65% ND services 59% Nutrition counseling 50% Massage therapy 47%
Frequency of services	Manual and exercise therapy (with chiropractors) 2×/wk for 9 weeks 1×/wk for 4 weeks 1×/mo for two sessions for follow-up
	Psychological counseling/CBT 1×/wk for 10 weeks 1×/2 wk for two sessions In 1 month for follow-up
	ND and nutrition counseling 1×/2 wk for two sessions (for each discipline) 1×/mo for two sessions (for each discipline)
	Massage therapy 1×/wk for 10–12 weeks Mindfulness class 2.5 h 1×/wk for 11 weeks (or one-to-one × 10 sessions)

^a^Each patient who completes the program receives 65–80 h of one-to-one services including participation in our mindfulness program in his or her 3- to 4-month journey.

PWC = Pain & Wellness Centre; CBT = cognitive behavioral therapy; ND = naturopathic doctor.

Interdisciplinary communication between members of the treating team is intense and frequent and includes formal IDP rounds where selected patients are discussed with all providers and physicians, internal speedy communication using Slack (an electronic internal communication medium), access to all electronic medical records that must be uploaded by each provider within 48 h, and “informal knock on the door” (“open door policy”).

Upon discharge (exit) from the program, patients complete questionnaires (identical to those at the start of the program) plus Global Impression of Change scales. These questionnaires are provided again at 6 and 12 months postdischarge from the IDP. During their participation in the program, patients are reviewed periodically as needed by the medical staff; medical follow-up appointments are made as well at 6 and 12 months after completion of the program to assess progress and address any problems, with the patient’s evolution and progress documented and communicated at each medical appointment to his or her referring physician. Patients can be granted additional hours of treatment service based on the recommendation of their treating team, particularly for support during return to work, when changing jobs, or when some unexpected issue arises (interruption by hospitalization, family emergency, acute injury, etc.). Patients who miss two to three sessions without a justified reason are seen by the medical staff to discuss the reasons for their absenteeism, and those who continue to miss sessions are dismissed from the program or fail to return on their own.

A key aim of our program is a move toward a holistic and coordinated model. Patient-centered care is central and relationship based.^15^ It is clearly explained in the IDP consent form with the following verbatim statement: “I understand that I am part of the team and that I must take an active role in regaining control of my pain and my life. I understand that each member of the pain team will work closely with me to set specific goals, provide tools and techniques that I can use on my own and monitor my progress.” It invites therapeutic balance and highlights the centrality of an informed person choosing relevant strategies.

Since our funding began as of April 1, 2016, we have admitted (up to October 23, 2018) 242 patients to the IDP program and offered 13 977 h of treatments by allied health providers. Of those 242 patients, 37 (16%) dropped out (preliminary data were presented as a poster at the 2018 Canadian Pain Society meeting), 36 completed only our demographic questionnaire, outcome exit data exist for 123 patients, and the rest are still in treatment. Overall, it is estimated that each IDP patient receives 60 to 85 h of primarily one-to-one treatments.

### Education and research output

The PWC is recognized by the University of Toronto Pain Medicine as a site of compulsory rotation for all pain medicine residents, who have obtained a Royal College of Physicians and Surgeons (RCPS) certification in a number of specialties such as anesthesia or physical medicine and pursue a further 2-year residency training in noninterventional pain management and further RCPS certification in the subspecialty of pain medicine.

Additionally, the PWC shares pain fellows with the Toronto Rehabilitation Institute (TRI) and serves as a site of elective rotation of physical medicine residents during their “pain block.” University of Toronto family practice residents and Canadian Memorial Chiropractic College residents and interns approach us directly for pain electives. The PWC frequently hosts teams of practicing physicians and allied health professionals for half-day visits on-site to observe our operations. We further provide continuous health education sessions for the local physicians and occasional lectures to the public (patients and families).

**Figure 2.**
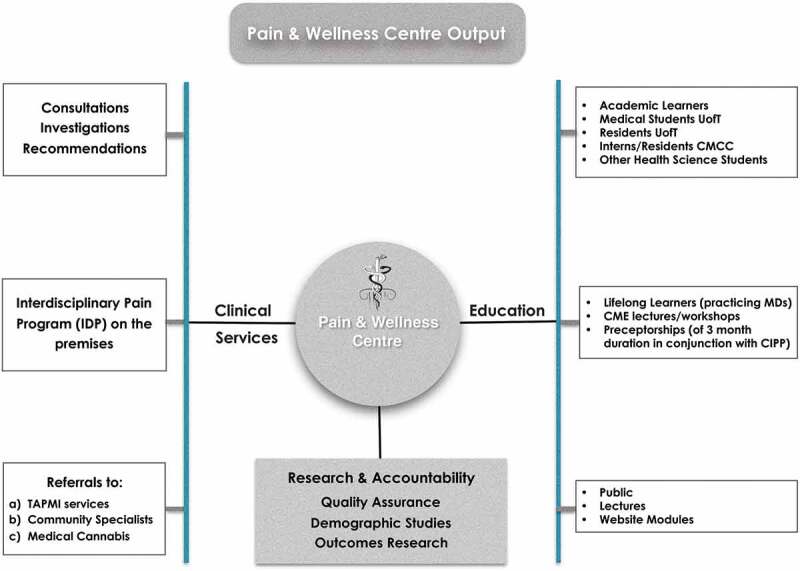
The PWC outputs.

In regard to research, we employ a senior research associate, assisted by students for data synthesis and analysis and some of our administrative staff for data collection, access to the research ethics board of the University of Toronto, organization of our research studies (retrospective and prospective), and grant submissions. Our data are mined partially through our electronic medical records system and primarily through separate databases maintained by our administrative and research staff. The PWC output in regard to clinical services, education, and research/accountability metrics is shown in Figure 2.

### Integration with community resources and patients

The PWC has formed alliances with local facilities as follows: an x-ray facility that performs the majority of our radiological investigations in a timely manner; a neurologist for all of our electrophysiological testing; an interventional facility for all of our interventions and infusions; and a compounding pharmacy for our compound prescriptions.

Additionally, the PWC has forged close liaisons with a large multispecialty community-based facility for exchange, referral, and investigations of patients with suspected inflammatory and autoimmune disorders. We have also established links with a specific nearby medical cannabis clinic, where we refer eligible patients.

We have further created the unique position of a community resource facilitator who collected ledgers of community resources that may be available to our patients for navigation of services (e.g., lists of therapeutic pools, social services, mental health services, medical cannabis clinics, etc.). She also works one on one with those patients who require assistance in initiating community supports and/or resources. Additionally, our community resource facilitator is charged with visiting family health teams and individual practices to inform and educate them in regard to our services, our criteria for proper referrals, and asking for early referrals of pain patients, so that chronicity can be curtailed.

The director of the PWC and senior author has served as chair of a not-for-profit patient organization (ACTION Ontario) since 2005 and continues to be formally involved in the publication of their newsletters, connecting PWC patients with patient members of ACTION, reaching out to ACTION members and PWC patients for guidance relating to clinic and MOHLTC initiatives, and integrating patients’ input into the PWC’s operations.

### Relationship with the Toronto Academic Pain Medicine Institute

The PWC is associated bidirectionally with Toronto Academic Pain Medicine Institute (TAPMI)-associated hospitals (a concerted and organized network of five Toronto downtown hospitals funded by MOHLTC) in regard to education of our trainees (described earlier).

In terms of clinical services, the PWC refers a number of our patients to TAPMI-associated hospitals for interventions, spinal stimulators, total joint replacements, etc.; accepts to the IDP certain referrals of patients from TAPMI; and selected PWC patients are admitted to the TRI/University Health Network inpatient beds for interdisciplinary investigations under TRI staff. Inpatient TRI rounds are attended by PWC medical staff via video teleconferencing.

### Preliminary results of system change

An abstract presented at the Canadian Pain Society meeting^16^ reported on outcome data of 47 patients who had exited our program at 3 months, indicating substantial improvement in several validated scales (60.2% in self-efficacy, 48.8% in pain catastrophizing, 49.8% in GAD, 41.5% in BPI, 31.2% in Centre for Epidemiological Studies–Depression Scale), with 83% of the participants much/very much improved in Global Impressions of Change. In regard to demographics characteristics, the male : female ratio was 1:2 (*P* < 0.5); mean age 47 ± 18 years (18–85 years); Canadian-born patients constituted 65% (*P* < 0.05); 44% were employed; 30% consumed marijuana over the past year; and one third had received multiple injections in the past. In 50% of patients, pain originated from motor vehicle accident, work, and sports injuries. Mean pain ratings and pain duration were 6.1 ± 1.6 and 5 ± 6 years, respectively.

A follow-up abstract has been submitted and accepted for presentation to the upcoming Canadian Pain Society 2019 meeting with a larger cohort of 121 patients treated through the PWC IDP. These data show that the much/very much improved rate on Global Impressions of Change continues to stand at 79% at the 3-month exit point and preliminary data at 6 and 12 months continue to demonstrate high levels of sustained success. The poster to be presented at the April 2019 meeting will include a more comprehensive analysis of changes from the point of entry to the point of 3-month exit, as well as longer term outcome data.

So far, our data have or are being presented in 11 posters and several oral presentations, in four local meetings, in three national conferences, and in one international conference, and we are in the process of submitting several papers for peer-reviewed publications.

In subsequent papers we plan to report on our results including (but not limited to) characteristics of responders and nonresponders to our IDP, costs of the IDP program, return to work/school and other outcomes, opioid consumption of new patients referred to the center, morphine-equivalent dose in subgroups (based on age, gender, diagnosis, and ethnicity), cannabis past and current use, opioid use and cannabis use in the subset of patients treated in our IDP, etc.

### Future goals

The PWC expects to continue its work offering consultation services and interdisciplinary pain management in eligible chronic pain patients in the community.

In the current year we expect to submit/publish in peer-reviewed journals the observed outcomes from our IDP, as well as the results of cross-sectional descriptive studies that describe the community populations that attend our center.

The PWC director will continue to participate actively in MOHLTC committees and initiatives to assist the MOHLTC adult and pediatric networks.

The PWC aims to forge further links with community primary care and specialist groups to promote evidence-based chronic pain diagnosis and management.

Furthermore, the PWC is interested in designing and participating in pragmatic community-based studies in issues that matter (e.g., management of opioid misuses; risks, benefits, and effectiveness of medical cannabis in our population; collaborative studies of Virtual Reality applications in our chronic pain population, etc.).

## Conclusions

This brief overview of the 2.5 years of operation of the first Ontario community-based pain clinic supported by public funds confirms that the PWC model fulfills nearly all of the principles of the Ontario comprehensive pain strategy. It is interdisciplinary and multimodal; patient centered; primary care focused; operating within the chronic disease management framework; providing evidence-based and stepped-up care; accountable to MOHLTC in regard to the services paid by taxpayer’s funds and the outcomes of these services; consistent; providing access to prevention and early intervention; providing education for both patients and health providers; showing continuous quality improvement; supporting data information exchange and research; and offering reasonable access to the care continuum. Additionally, it provides navigation of the complex health care system to patients with chronic pain in our community with chronic pain, and it integrates patient input in important matters.

None of the above could have materialized if it were not for sustainable funding provided by MOHLTC. Ultimately and inevitably, funding influences service delivery, because many patients cannot afford the necessary interdisciplinary (nonmedical) services. The described patient-centered PWC model for chronic pain does improve engagement with the individual with chronic pain and offers a viable model of pain care delivery in the community. In this context, PWC has fulfilled the expectation of MOHLTC for providing a model and a template for similar community-based and publicly funded pain clinics.

However, a major gap remains in the present PWC funding model regarding the FFS remuneration of pain physicians, because the model strictly provides funding only for nonmedical services. Since the 2011 Ontario Comprehensive Chronic Pain Strategy Report, the Royal College of Physicians and Surgeons of Canada has recognized the subspecialty of pain medicine^24^. Despite the fact that there are now new, well-trained graduate physicians, RCPS certified in multimodal interdisciplinary pain medicine, the FFS model will make non-intervention-based medical chronic pain care delivery unsustainable, because chronic complex pain patients require lengthy consultations and longitudinal care. Hybrid models of medical remuneration (including a combination of FFS, alternative pain funding, management fees, etc.) are necessary.

A further word of caution: Intense interdisciplinary pain management is not suitable for every patient with chronic pain because public funding is not unlimited. Our selection criteria that place emphasis on patient motivation and commitment are very important in stratifying patients and selecting those who are more likely to improve and capitalize on the interdisciplinary resources of pain management provided to them through public funds. Less resource-intense programs such as group self-management education, group classes (like Tai Chi), web resources, etc., may fill the gap for some patients. Detailed medical consultations can assist with diagnosis, provide proper pharmacology, and offer advice to referring physicians, as well as initiate entry to our IDP for eligible patients, while judicious and careful use of interventions, spinal stimulators, and surgeries can also have their place in well selected chronic pain patients.

Based on our experience and that of the literature, the gap between effort and progress in chronic pain care in general can be bridged through more effective engagement of patients in care processes and care goals. The solution lies in a truly patient-centered approach that engages the patient as the agent through which health care is delivered and by which health is achieved. There is no doubt that various jurisdictions, organizations, and health care systems are interested in moving toward a more person‐centered approach as systems rethink the way in which pain care is provided.^17–19^ Analysis of literature highlights four common dimensions in patient-centered care: biopsychosocial perspective; patient as person; sharing power and responsibility; and therapeutic alliance.^20^ Patient-centered care identifies with understanding of the illness or pain condition from the person’s point of view and seeing the person as a whole instead of as fragmented parts.^21^ An approach that is centered on the individual does not imply an individual’s unrestricted freedom of choice; rather, decision making is shared.

Looking at the larger picture, chronic pain management remains a provincial jurisdiction with gross inconsistencies and differences between provinces. Despite the Ontario comprehensive strategy and the progress obtained in Ontario through public funding, much remains to be done. There are continuous gaps in Ontario (and in Canada in general) in the health system, limiting availability and access to chronic pain programs and services. No single institution in Canada is charged with organizing the numerous aspects of pain management; there is no system for coordinating action, sharing learning, and distributing best practices to policymakers, health professionals, patients, and the network at large; and planning and care are fragmented, prompting a superfluous duplication of efforts and resulting in inefficient use of resources. This was highlighted recently in the evidence brief from the 2017 McMaster Forum, calling for the development of a national pain strategy.^22^

Though health care remains under provincial jurisdiction, the need for a national pain strategy is indeed pressing.^23^ The prevalent paradigm in Canadian pain medicine is under pressure, the need for evolution is clear, and the pivotal issue is eloquently stated in the McMaster’s evidence brief:

While there may be significant costs associated with preventing and managing chronic pain (many of which are not covered by provincial health insurance plans), failing to do so using evidence-based approaches may incur even greater costs. The money being spent to address the recent rise in illicit opioid-related morbidity and mortality is to some degree an example of such greater costs (Evidence Brief Developing a National Pain Strategy for Canada Dec 14 2017).
